# Supplementary material for: Reconfiguring Pain Interpretation Within a Social Model of Health Using a Simplified Version of Wilber’s All Quadrant All Levels Framework: An Integral Vision
Source: Behav Sci (Basel). 2025 May 20;15(5):703. doi: 10.3390/bs15050703 (PMC12109466; doi:10.3390/bs15050703)
Supplement: Supplementary file 1 [file behavsci-15-00703-s001.zip › behavsci-3553525-supplementary.pdf]

Reconfiguring Pain Interpretation Within a Social Model of Health Using a Simplified Version of Wilber's All Quadrant All Levels Framework: An Integral Vision

Mark I. Johnson

Supplementary Materials

Contents

Table S1. Pain mapped into the individual-interior quadrant. 2
Table S2. Pain mapped into the individual-exterior quadrant. 3
Table S3. Pain mapped into the collective-interior quadrant. 4
Table S4. Pain mapped into the collective-exterior quadrant. 5
Table S5. Pain and sub-categories of Wilber's transpersonal level. 6

Table S1. Pain mapped into the individual-interior quadrant.

Item	Features
The Individual's Experience	• Sensory-discriminative experience of pain: The characteristics of pain itself, such as spatial (location, breadth and depth) and temporal dimensions (constant, cyclical, intermittent, flare-ups), intensity.<br>• Affective, emotional-motivational experience of pain: Feelings of unpleasantness, anxiety, fear, frustration, depression and desire to act.<br>• Cognitive-evaluative experience of pain: Thoughts, beliefs and appraisals about pain, such as cause, perceived threat, expectation of prognosis, catastrophising.
Impact on the Individual's Health and Well-being	• Sensory Impact: How pain affects body percept (e.g., attractive or unattractive, strong or weak etc.) and schema (e.g., capable or unable, employable or unemployable etc.)<br>• Emotional Impact: How pain affects emotional well-being, such as psychological distress, emotional suffering (fear, anxiety, sadness, anguish, guilt, shame) and reduced desire to do things leading to social isolation.<br>• Cognitive Impact: How pain affects thoughts such as the cause of pain and associated the suffering, its implications on daily functioning, ability to cope, self-efficacy, sense of self and long-term future.<br>• Resilience and Coping: The inner resources individuals draw upon to live with pain, including psychological resilience, coping strategies, use of support services, and interventions for pain management.
Interventions	• Psychologically interventions that focus on an individual's inner state such as cognitive-behavioural therapy (CBT), acceptance and commitment therapy (ACT),<br>• mindfulness, meditation, positive psychology, compassion-focussed therapy, psychotherapy, counselling, meditation, art therapy, engagement with arts.
Body of knowledge	• Research on the psychology of pain is extensive and robust. Research on first-person experience of pain is substantial and diverse and is gaining prominence in healthcare.
Influential sector	• The public and private healthcare sector provides services, treatments, and care to individual's to alleviate suffering and improve quality of life for individual's in pain. Various healthcare practitioners support the psychology of individuals in pain, especially those with complex needs, such as psychologists, psychotherapists and allied health professionals. The healthcare sector considers the VCSE sector a secondary support service for care of the self.
Opportunities to transform perspective	• Exploring pain and psychophysiological 'dis-ease' in the context of their inner-self may assist an individual<br>o Reconceptualise understanding of pain including the importance of psychological factors.<br>o Reshape a meaningful sense of self as a human experiencing pain as part of a journey of being and becoming.<br>o Reveal new ways to express and communicate pain<br>• Engagement with art and creativity and culturally-adapted pain education

Table S2. Pain mapped into the individual-exterior quadrant.

Item	Features
The Individual's physiology	• This includes observable structural and functional changes in body tissue in the immediate, short and longer-term including influencing the emergence of pain and associated physiological responses and the outward expression of pain and associated behaviour. For example, nociception, sensitisation, neuromodulation, bioplasticity, maladapted neurophysiology, and responses of other body systems (e.g., HPA-axis mediated immune, cardiorespiratory etc.),
The Individual's outward behaviour	• This includes how pain affects an individual's behavioural responses, such as changes in mobility, and posture, daily functioning and participation in activities and disability; and coping strategies, such as modifications in lifestyle, use of assistive devices, and engagement in pain management techniques; and how individuals interact with healthcare providers (e.g., options, availability and choice of services, therapy shopping) and behaviours associated with treatment plans and regimens, including medication use, physiotherapy, and other interventions.
Interventions	• Tissue-targeting interventions to repair and relieve (e.g., drugs, surgery, neuromodulation, drugs, massage, electrophysical agents) and assist functioning (e.g., walking aids). Psychological interventions target thoughts and emotions to influence behaviour (e.g., CBT, ACT, supported-self management)
Body of knowledge	• Research on the bioscience of pain is extensive and robust, and the vast quantities of biomedical clinical and scientific studies dominates knowledge from other quadrants and governs societal narratives about pain and its treatment. There are concerns that some clinical research lacks rigor.
Influential sector	• The public and private healthcare sector provides services, treatments, and care to individual's to alleviate suffering and improve quality of life for individuals in pain. Various healthcare practitioners treatment the body such as physicians, physiotherapists, nurses, chiropractors. Concerns about overmedicalisation of pain and market forces driving the health care (medical), and bioscience private and public industries. The healthcare sector considers the VCSE sector a secondary support service for care of the self.
Opportunities to transform perspective	• Advancing biomedical knowledge towards individualised precision tissue-based diagnosis (e.g., biomarkers) and treatments.<br>• Reconfiguring the dominance of the biomedical paradigm to assist an integral understanding of pain.

Table S3. Pain mapped into the collective-interior quadrant.

Item	Features
Collective attitudes and norms	• The collective values attributed to pain that shape cultural norms about how to experience, express and respond to pain. Influences include upbringing, socialisation, communication abilities, power dynamics, stigma associated with pain, the legitimacy of pain, and the types of services and support considered acceptable.
Collective narratives	• This includes cultural narrative and worldview about pain that impacts how individuals and communities understand and address pain e.g., a religious or medicalised meaning for pain.
Interventions	• Interventions targeting the intersubjective space of shared experiences and mutual understandings of pain. This may include culturally-adapted interventions to assist communities and individuals explore pain narrative and possibilities to reconceptualise pain through pain education, CBT, ACT.
Body of knowledge	• Scholarship on culture, morals and ethics of pain is longstanding, substantial and diverse, although primary research on the sociology, culture and societal attitudes of pain is often obscured by biomedical research. In recent years, there has been increasing attention about the intersubjective aspects of pain.
Influential sector	• The healthcare sector carries the societal narrative and culture of pain, strongly influenced by governments and corporate industry (e.g., pharma, medical devices, instruments and gadgets). Religion also has influence.
Opportunities to transform perspective	• Greater prominence to the social model of pain to reduce stigma, injustices and inequalities associated with social determinants of pain.<br>• Reshaping societal mindset from pathogenic to salutogenic using constructive pain language and health promotion strategies.

Table S4. Pain mapped into the collective-exterior quadrant.

Item	Features
Collective Environment	• This includes how global, national, regional, and local level systems and structures<br>• affect individuals with pain. For example, the influence of physical conditions of housing or workplaces, safety, costs of living, discrimination, social integration, and quality of social services.
Collective policy and regulation	• This includes how policies, regulations, and laws impact allocation of resources for social service provision, treatment and community-based support for pain, such as policies related to pain medications, disability benefits, and patient rights.
Interventions	• Social and environment-targeting interventions that affect the accessibility and quality of healthcare services available for managing pain such as healthcare infrastructure, insurance coverage, and the availability of pain services and support, both specialized and non-specialised.
Body of knowledge	• Research and scholarship on the environment and pain is modest but growing. Knowledge about the influence of social, economic, and environmental factors on pain is informed by epidemiology, of which there is substantial evidence, and sociology, public health, and health policy.
Influential sector	• The healthcare sector is seen as the main 'setting' and influenced by government, financial, and business sectors. Other influential sectors include media and technological, and religion.
Opportunities to transform perspective	• Exploration of insidious macro level 'forces' within present day socio-ecological habits (systems and structures) that prime severity and stickiness of pain and associated suffering – i.e., the painogenic environment.<br>• Development of health policies and strategies to foster 'healthy settings' to create supportive ecologically concordant urban design and community empowerment to reduce inequalities and encourage healthy attitudes and behaviours towards pain.

Table S5. Pain and sub-categories of Wilber's transpersonal level.

Wilber describes sub-categories of transpersonal development where individuals see connections between systems (vision-logic) and progress through psychic, subtle, causal, and nondual levels, marked by deeper spiritual awareness, the dissolution of the ego, and the realization of the oneness of all existence. In the sub-category Vision-Logic Level, pain is seen within an interconnected system, combining medical, psychological, and spiritual practices. The Psychic Level brings heightened intuition, viewing pain as an energetic disturbance addressed through energy work and meditation. The Subtle Level sees pain as deeper emotional or energetic issues addressed by somatic practices like yoga and acupuncture. The Causal Level views pain as a temporary sensation managed by deep meditation and mindfulness. The Non-Dual Level understands pain as part of existence, emphasizing non-duality, compassion, and acceptance.

Transpersonal sub-category	Characteristics	Experience of Pain	Coping	Example
Vision-Logic Level	Integration of rational thought with a holistic understanding of reality with an ability to see patterns and connections across different domains.	Pain is understood within a broader, interconnected system and seen not just as a physical sensation but also as an emotional need state influenced by biopsychosocial factors.	Holistic approaches that combine medical treatments with psychological and spiritual practices that address pain through lifestyle changes, stress management, and understanding underlying causes.	
Psychic Level	Heightened intuition and psychic experiences with greater sensitivity to subtle energies and deeper emotional experiences.	A deeper emotional and intuitive understanding of pain that may be identified as an 'energetic' disturbance or imbalance.	Reiki, acupuncture, and other forms of energy work to complement meditation, visualization, and inner work to understand and heal pain	
Subtle Level	This level involves a deeper exploration of the subtle aspects of consciousness, including energy and emotional states.	The individual may recognise pain as a manifestation of deeper emotional or 'energetic issues', leading to a more holistic knowing of their suffering. Pain may be viewed as an opportunity for inner work and transformation.	Utilizing somatic practices, or bodywork, with non-medicalised, and less conventional explanatory narratives, such as chakras, energy work, and meridians, such as yoga, Reiki, traditional Chinese acupuncture to release, form example 'energy' or 'emotional	An individual that engages in somatic experiencing or energy healing to explore the emotional roots of their pain, seeking to release trauma stored in the body.

			blockages' associated with long-term pain	
Causal Level	This level involves the realization of deeper states of consciousness, often associated with profound inner peace and stillness.	The individual recognises pain as a temporary sensation that does not define their being, understanding that pain is not necessarily physical (a thing of stuff) but part of the impermanent nature of existence; facilitating a profound sense of detachment from their pain whilst at the same time recognizing it.	Engaging in deep meditation, mindfulness practices, or transcendental experiences that cultivate a sense of unity with all beings and a profound acceptance of suffering.	A person uses advanced meditation techniques to achieve a state of non-attachment to their long-term pain, experiencing moments of bliss and profound acceptance despite physical discomfort.
Non-Dual Level	This highest level represents the realization of non-duality, where individuals experience a fundamental unity of all existence and transcend the duality of subject-object.	The individual recognises pain as part of the totality of existence, seen through the lens of unity and interconnectedness. The individual recognizes that their identity and pain are not separate from the universe; instead, they are expressions of the same consciousness.	Practices that emphasize non-duality and 'oneness', such as advanced spiritual inquiry, contemplative awareness, or deep presence. Individuals may cultivate compassion for themselves and others as integral to human experience.	An individual experiences moments of profound unity with their pain, viewing it as an expression of life itself, leading to a deep sense of peace and acceptance.
